# Purification and Biochemical Characterization of a Novel Fibrinolytic Enzyme from Culture Supernatant of *Coprinus comatus*

**DOI:** 10.3390/foods13091292

**Published:** 2024-04-23

**Authors:** Jinyu Wang, Xiaolan Liu, Yan Jing, Xiqun Zheng

**Affiliations:** 1 College of Food Engineering, Harbin University of Commerce, Harbin 150076, China; 02758@qqhru.edu.cn; 2 Key Laboratory of Corn Deep Processing Theory and Technology of Heilongjiang Province, College of Food and Bioengineering, Qiqihar University, Qiqihar 161006, China; 02837@qqhru.edu.cn (Y.J.); zhengxiqun@byau.edu.cn (X.Z.); 3 College of Food, Heilongjiang Bayi Agricultural University, Daqing 163319, China

**Keywords:** *Coprinus comatus*, fibrinolytic enzyme, anticoagulant activity, fermentation

## Abstract

A novel fibrinolytic enzyme was produced by the liquid fermentation of *Coprinus comatus*. The enzyme was purified from the culture supernatant by hydrophobic interactions, gel filtration, and ion exchange chromatographies. It was purified by 241.02-fold, with a specific activity of 3619 U/mg and a final yield of 10.02%. SDS-PAGE analysis confirmed the purity of the enzyme, showing a single band with a molecular weight of 19.5 kDa. The first nine amino acids of the N-terminal of the purified enzyme were A-T-Y-T-G-G-S-Q-T. The enzyme exhibited optimal activity at a temperature of 42 °C and pH 7.6. Its activity was significantly improved by Zn^2+^, K^+^, Ca^2+^, Mn^2+^, and Mg^2+^ while being inhibited by Fe^2+^, Fe^3+^, Al^2+^, and Ba^2+^. The activity of the enzyme was completely inhibited by ethylenediamine tetraacetic acid (EDTA), and it was also dose-dependently inhibited by phenylmethylsulfonyl fluoride (PMSF) and soy trypsin inhibitor (SBTI). However, inhibitors such as N-α-tosyl-L-phenylalanine chloromethyl ketone (TPCK), aprotinin, and pepstatin did not significantly affect its activity, suggesting that the enzyme was a serine-like metalloproteinase. The enzyme acted as both a plasmin-like fibrinolytic enzyme and a plasminogen activator, and it also exhibited the capability to hydrolyze fibrinogen and fibrin. In vitro, it demonstrated the ability to dissolve blood clots and exhibit anticoagulant properties. Furthermore, it was found that the enzyme prolonged activated partial thromboplastin time (APTT), prothrombin time (PT), and thrombin time (TT), and reduced the levels of fibrinogen (FIB) and prothrombin activity (PA). Based on these studies, the enzyme has great potential to be developed as a natural agent for the prevention and treatment of thrombotic diseases.

## 1. Introduction

The incidence and mortality of thromboembolic diseases have been increasing year by year, making it a major threat to human health. Thrombosis is a complex physiological process involving fibrinogen, thrombin, coagulation factors, and coagulation inhibitors [1]. Insoluble fibrin can be hydrolyzed to fibrin degradation products (FDPs) by fibrinolytic enzymes. Under normal physiological conditions, the fibrinolytic and clotting systems in the body maintain a dynamic equilibrium [2,3]. However, in a state such as disease or aging, the dynamic balance is disrupted. In the abnormal fibrinolytic system, fibrinogen forms fibrin under the action of thrombin and polymerizes into fibrin clots in the blood vessels, leading to thrombosis [4].

Fibrinolytic enzymes are a kind of proteolytic enzyme. Fibrinolytic enzymes can be classified into plasminogen activators and plasmin-like proteases, based on the mechanism of fibrinolysis. Plasminogen activators activate plasminogen into plasmin, whereas plasmin-like proteases can degrade fibrin clots directly [5,6]. At present, the main clinical plasminogen activators are streptokinase, tissue plasminogen activator (t-PA), and urokinase plasminogen activator (u-PA). Despite being in widespread use, these reagents have drawbacks such as a short half-life, low specificity for fibrin, high price, and excessive bleeding [7,8]. Therefore, the exploration and development of a safe and natural source for new thrombolytic agents with fewer or no side effects have become the key to the prevention and treatment of cardiovascular diseases.

Microbial fermentation is the main method for producing fibrinolytic enzymes, and it allows for the rapid production of a large quantity of target products. Over the past decade, there has been a significant amount of research conducted on fibrinolytic enzymes that are extracted from various fermented foods with traditional Asian origins [9,10]. New strains need to be developed for fermentation to produce fibrinolytic enzymes. Edible mushrooms grow in a wide regional range, often used as food, traditional Chinese medicine, or folk medicine [11]. Mushrooms are rich sources of natural bioactive compounds, such as polysaccharides, fibrinolytic enzymes, and phenolic compounds, which have become a hot topic for research. It has been found that fibrinolytic enzymes can be isolated from the fruiting bodies of edible mushrooms, mycelium, and fermentation broth [12,13,14,15,16].

*Coprinus comatus*, a traditional Chinese medicinal mushroom, is mainly distributed in temperate and subtropical humid areas [17]. It possesses abundant nutritional value and a substantial protein composition, containing 20 different types of amino acids, including 8 essential ones, as well as some bioactive components. Studies have found that it has several physiological functions such as antioxidant effects, hypoglycemic effects, prevention of liver damage, anti-tumor effects, immunity enhancement, and bacteriological inhibition [18]. Currently, the majority of research on *Coprinus comatus* primarily focuses on its capacity and functionality in polysaccharide production [17,18,19]. Prior to our current report, in the literature available, a fibrinolytic enzyme from *Coprinus comatus* has not been reported. In this preliminary study, the culture conditions for the liquid fermentation of the fibrinolytic enzyme from *Coprinus comatus* (CFE) were optimized and the productivity improved significantly. The objective of the present study is to purify the fibrinolytic enzyme from the fermentation supernatant of *Coprinus comatus* and study its properties, as well as its anticoagulant and thrombolytic activities.

## 2. Materials and Methods

### 2.1. Materials

Octyl-Sepharose Fast Flow, Sephadex G-25, SP-Sepharose High Performance, and Source 15PHE were purchased from GE Life Sciences (Pittsburgh, PA, USA). Fibrinogen, thrombin (bovine and human source), immunoglobulin G, and human serum albumin (Shanghai Jingke Biological Co., Ltd., Shanghai, China). Ammonium sulfate, agarose, a low-molecular-weight protein standard kit for SDS-PAGE, and sodium chloride (Sangon Biotech Co., Ltd., Shanghai, China). Soybean trypsin inhibitor (SBTI), N-α-tosyl-L-phenylalanine chloromethyl ketone (TPCK), phenylmethylsulfonyl fluoride (PMSF), aprotinin, pepsin, and pepstatin (Sangon Biotech Co., Ltd., Shanghai, China). Coomassie Brilliant Blue R-250 (Sangon Biotech Co., Ltd., Shanghai, China). KCl, NaCl, NaHCO_3_, CaCl_2_, and HCl (Kemi Ou Chemical Reagent Co., Ltd., Tianjin, China).

### 2.2. Strain and Culture Conditions

The *Coprinus comatus* strain was stored on PDA slants. The mycelia were transferred from the slants to the plate medium. Aseptic inoculation of the mycelia was performed in 250 mL shake flasks, each containing 50 mL of fermentation media composed of 2.9% fructose, 3.6% soybean cake, 0.25% KH_2_PO_4_, and 0.2% MgSO_4_. The fermentation process was conducted at a temperature of 24 °C and a speed of 160 r/min for 6 days. After that, the resulting mixture underwent centrifugation at a rate of 10,000 r/min for 10 min at 4 °C. The resulting supernatant was then considered the crude enzyme extract.

### 2.3. Purification of the Fibrinolytic Enzyme from Coprinus comatus

The crude enzyme was subjected to 60% saturation of ammonium sulfate precipitation and stored at 4 °C for 12 h. The supernatant, obtained by centrifugation (8000 r/min for 20 min at 4 °C), was dissolved with 20 mM sodium phosphate buffer (pH 7.4). The enzyme solution was loaded onto an Octyl-Sepharose FF column (1.6 cm × 30 cm) which had previously been equilibrated with 20 mM sodium phosphate buffer (pH 7.4) containing 30% saturated (NH_4_)_2_SO_4_. Bound proteins were eluted with a decreasing linear gradient of 30–0% (NH_4_)_2_SO_4_ in 20 mM sodium phosphate buffer (pH 7.4). Each tube collected 6 mL of the active component. The buffer was exchanged on a Sephadex G—25 column (1.6 cm × 80 cm) using 20 mM sodium phosphate buffer, pH 4.0. The enzyme preparation, after desalting, was loaded onto an SP-Sepharose HP column that had been pre-equilibrated with the same buffer. Elution was performed using a linear gradient of NaCl concentration ranging from 0 to 0.1 M. Each tube collected 4 mL of the active component. Fractions with the highest potency were combined and subjected to further purification using a Source 15PHE column. The column was initially equilibrated with a sodium phosphate buffer (pH 7.4) containing 20% saturated (NH_4_)_2_SO_4_, followed by elution using a linear gradient of ammonium sulfate ranging from 20% to 0% in the same buffer. Each tube collected 2 mL of the active component. Finally, the purified active fractions were pooled, lyophilized, and used for further characterization.

### 2.4. Fibrinolytic Enzyme Activity Assay

Fibrinolytic enzyme activity was estimated according to the improved method of Astrup and Mullertz [20]. The fibrin plates were prepared according to Liu et al. [13]. Fibrinolytic enzyme activity was assessed using the fibrin plate method with urokinase as the reference standard.

The protein concentration of the samples was quantified by the Lowry method [21]. The standard protein content curve was prepared using bovine serum albumin (BSA) as the reference protein, with the standard protein serving as the independent variable and the absorption value at OD640 nm as the dependent variable. The regression equation y = 0.0023x + 0.0114 was derived.

### 2.5. Determination of Molecular Weight and Fibrin Zymography

The SDS-PAGE method, as described by Laemmli [22], was employed to assess the molecular weight and purity of the purified fibrinolytic enzyme. The molecular weight of standard proteins ranges from 10 to 170 kDa. The fibrin zymography of CFE was detected according to Li [23]. The resolving gel solution, which contained 0.12% (*w*/*v*) fibrinogen and thrombin (2 U/mL), was prepared. After electrophoresis of the purified enzyme on the fibrin gel, it was soaked in a 2.5% Triton X-100 solution for 30 min and incubated overnight at 37 °C in a reaction buffer bath (20 mM Tris–HCl buffer, pH 7.4, containing 0.15 M NaCl). The gel was washed with distilled water and then stained with Coomassie Brilliant Blue R-250. The digested bands were visualized as non-stained regions on the fibrin gel.

### 2.6. Determination of the N-Terminal Amino Acid Sequence

The Edman degradation method was employed to determine the N-terminal amino acid sequence of CFE. Following purification, the enzyme underwent analysis using SDS-PAGE and Coomassie Brilliant Blue R-250 staining. Subsequently, an electroblotting system facilitated the transfer of protein bands onto a polyvinylidene difluoride (PVDF) membrane. The stained band was used for N-terminal sequencing at Applied Protein Technology (Shanghai, China).

### 2.7. Effects of Temperature and pH on the Activity of CFE

The purified enzyme (105 U/mL) was incubated on the blood fiber plate for 6 h within a temperature range of 22 °C to 67 °C. For thermostability, CFE was incubated at different temperatures ranging from 24 °C to 57 °C for 1 h, 2 h, and 4 h. Subsequently, the residual fibrinolytic activity was measured by the standard fibrin plate method. The optimal pH of the purified enzyme was determined at 37 °C in 20 mM of different buffers (pH 2.8–12.4). For pH stability, CFE was incubated at 37 °C for 2 h, 4 h, 6 h, and 24 h in a range of pH buffers, and the residual fibrinolytic activity was determined.

### 2.8. Effects of Metal Ions, Protease Inhibitors, and Some Other Reagents on the Enzyme Activity

The effect of various metal ions (K^+^, Cu^2+^, Na^+^, Mg^2+^, Zn^2+^, Fe^2+^, Fe^3+^, Ca^2+^, Ba^2+^, Mn^2+^, and Al^2+^) at a concentration of 5 mM on enzyme activity was investigated. The metal ions and enzymes were incubated together at 37 °C for 12 h, followed by measurement of residual enzyme activity.

The effects of different protease inhibitors (ethylene diamine tetraacetic acid (EDTA), phenylmethyl sulfonyl fluoride (PMSF), aprotinine, soybean trypsin inhibitor (SBTI), N-α-tosyl-L-phenyl alanine chloromethyl ketone (TPCK), and pepstatin) on the enzyme activity were studied. These inhibitors were mixed with an equal volume of CFE and incubated at concentrations ranging from 1.0 mM to 10 mM at 4 °C for 12 h.

The effects of some other reagents on the enzyme activity were also studied. CFE was incubated with various additives (20 mmol/L cysteine, 5 mmol/L reduced glutathione, 5 mmol/L oxidized glutathione, 0.5% β-mercaptoethanol, 1% peptone, 1% gelatin, and 1% bovine serum albumin), organic solvents (10% acetone, 10% glycerol), and denaturants (urea and SDS) at 4 °C for 12 h. The remaining enzyme activity was measured accordingly.

### 2.9. Determination of Plasminogen Activator Activity

The fibrin plate method was used to analyze the plasminogen activator activity of CFE. Generally, commercially available fibrinogen contains a limited quantity of plasminogen. The preparation method of plasminogen-positive plates remained consistent with the description in Section 2.4. Plasminogen-negative plates were heat-treated at 85 °C for 30 min to inactivate plasminogen, ensuring the plates were devoid of any residual plasminogen activity. A 10 μL volume of CFE was added to different plates and incubated at 37 °C for 6 h, with urokinase used as a control. The presence of plasminogen activator activity was determined by observing differences in the size and clarity of the lytic zone in plasminogen-negative and plasminogen-positive plates.

### 2.10. Effects of Simulated Blood and Gastric Environment on the Enzyme Activity

The blood simulation solution, used in mammalian heart perfusion tests, known as Locke solution, consisted of 0.042% KCl, 0.9% NaCl, 0.02% NaHCO_3_, 0.024% CaCl_2_, and 0.2% glucose and was stored at a temperature of 4 °C. The simulated gastric juice was prepared by autoclaving a 10% HCl solution (pH 2.0–3.0) at 121 °C for 15 min, followed by cooling to 37 °C and mixing with 1% pepsin. The fibrinolytic activity of CFE was tested in seven different combinations of simulated blood and artificial gastric juice (Table 1).

### 2.11. Analysis of Thrombin-like Activity of CFE

The thrombin-like activity of CFE was investigated by analyzing the formation of fibrin clots in different mixtures (Table 2). The positive control group was composed of a mixture containing human blood fibrinogen and human thrombin. The mixture of human blood fibrinogen (1 mL, 10 mg/mL) and CFE (500 μL, 72 U/mL) was placed in a test tube and incubated for 10 min to observe the formation of thrombosis. Subsequently, human thrombin (500 μL, 200 U/mL) was added to the mixture and thrombosis was observed once again. The other tube contained a combination of human blood fibrinogen and CFE, and the production of fibrin was observed.

### 2.12. Dissolution of Blood Clots by CFE In Vitro

The dissolution effect of CFE on blood clots was determined according to the method of Zhou et al. [24] with minor modifications. CFE (500 μL, 72 U/mL) was co-incubated with whole blood clots (0.5049 g, 0.5065 g, 0.5059 g) obtained from healthy volunteers at 37 °C for 24 h. The samples were centrifuged at 3000 r/min for 30 s at various time intervals (10 min, 20 min, 40 min, 1 h, 1.5 h, 2 h, 2.5 h, 3 h, 12 h, and 24 h), and the supernatant was discarded. The residual clot was weighed to determine the clot dissolution rate.

$$\mathrm{Clot}\;\mathrm{dissolution}\;\mathrm{rate}=[\frac{\left(\mathrm{clot}\;\mathrm{weight}\;\mathrm{before}\;\mathrm{dissolving}-\mathrm{clot}\;\mathrm{weight}\;\mathrm{after}\;\mathrm{dissolving}\right)}{\mathrm{clot}\;\mathrm{weight}\;\mathrm{before}\;\mathrm{dissolving}}]\times 100\%$$

### 2.13. Fibrin(ogen)olytic Activity of CFE

The fibrinogenolytic activity of the purified enzyme was estimated according to the method of Liu [13]. A 20 μL (20 mg/mL) volume of human blood fibrinogen and 20 μL (72 U/mL) of CFE were mixed and incubated at 37 °C. Samples were collected at different time intervals, specifically 1 min, 5 min, 15 min, 30 min, 1 h, 1.5 h, 2 h, 3 h, 4 h, and 5 h. Cleavage patterns of fibrinogen were analyzed by SDS-PAGE.

To analyze the fibrinolytic activity of CFE, 20 μL of 2% fibrinogen was mixed with 10 μL of thrombin and allowed to clot. After clot formation, 30 μL CFE (72 U/mL) was added and incubated at 37 °C for different time intervals (1 min, 5 min, 15 min, 30 min, 1 h, 2 h, 3 h, and 4 h). The cleavage patterns of fibrin were analyzed by SDS-PAGE.

### 2.14. Effects of CFE on Some Blood Proteins

The influence of CFE on specific protein components in the bloodstream was also examined. A 20 μL solution of CFE (72 U/mL) was combined with equal volumes of human thrombin (15 mg/mL), immunoglobulin G (IgG, 15 mg/mL), and human serum albumin (HSA, 15 mg/mL), respectively, followed by incubation at a temperature of 37 °C for 4 h. Subsequent to incubation, the samples underwent analysis by SDS-PAGE.

### 2.15. Analysis of Anticoagulant Activity of CFE In Vitro

Platelet-poor plasma (PPP) was prepared from the blood of healthy volunteers (*n* = 6) by centrifuging the blood twice at 3000 r/min for 20 min at 4 °C, and the plasma in the supernatant was collected for use [25]. The reactants of different groups (Table 3) were incubated at 37 °C for 3 min. An automatic thrombin analyzer was used to determine five coagulation indexes in vitro, including prothrombin time (PT), fibrinogen content (FIB), activated partial thrombin time (APTT), thrombin time (TT), and prothrombin activity (PA).

The in vitro anticoagulant effect of CFE was also analyzed. Fresh human blood (1 mL) was mixed with CFE in sterilized centrifuge tubes. In separate tubes, the blood was mixed with normal saline (as a negative control) or heparin sodium (as a positive control). The experiment took place at 37 °C, and the process of blood clot formation was visually monitored continuously. To evaluate the anticoagulant activity, the tubes were inverted periodically every 5 s to observe any movement of blood along the tube and determine when coagulation occurred, indicating the external coagulation time.

## 3. Results and Discussion

### 3.1. Purification of a Fibrinolytic Enzyme from Culture Supernatant

**Figure 1:**
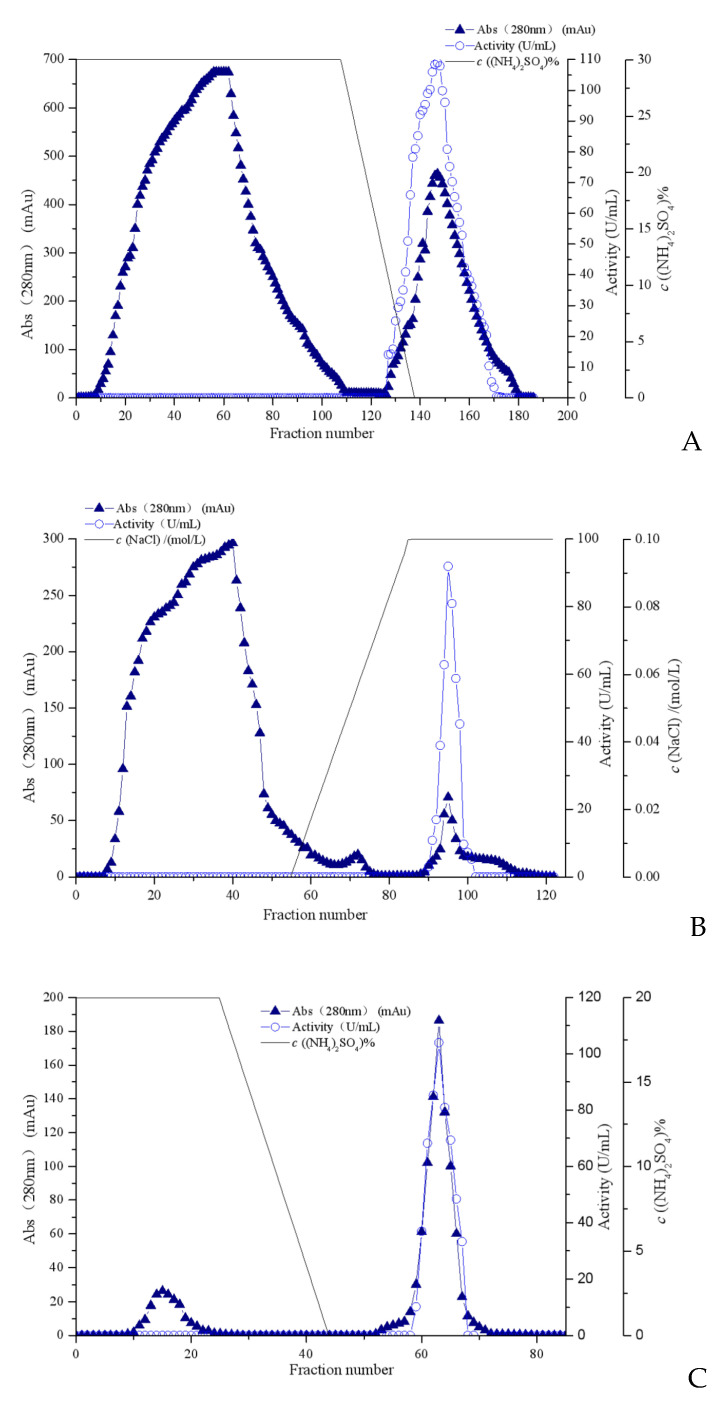
Purification of the fibrinolytic enzyme from *Coprinus comatus*. The elution profile of (**A**) Octyl-Sepharose Fast Flow hydrophobic interaction chromatography, (**B**) SP-Sepharose High Performance ion exchange chromatography, and (**C**) Source 15 PHE hydrophobic interaction chromatography.

The fibrinolytic enzyme was extracted from the supernatant of the liquid fermentation culture of *Coprinus comatus*. Under the optimized fermentation conditions, the enzyme activity reached 136.89 U/mL after fermentation for 6 days. The fermentation supernatant was collected as a crude enzyme solution. The fibrinolytic enzyme from *Coprinus comatus* was purified by ammonium sulfate precipitation, Octyl-Sepharose FF (Figure 1A), Sephadex G-25 column, SP-Sepharose HP (Figure 1B), and Source 15PHE (Figure 1C). The summaries of the recovery and purification folds for each step are shown in Table 4. The fibrinolytic enzyme from *Coprinus comatus* was purified by a fold of 241.02, with a specific activity of 3619 U/mg and an overall yield of 10.02%.

### 3.2. Molecular Weight and Purity Analysis of CFE

**Figure 2:**
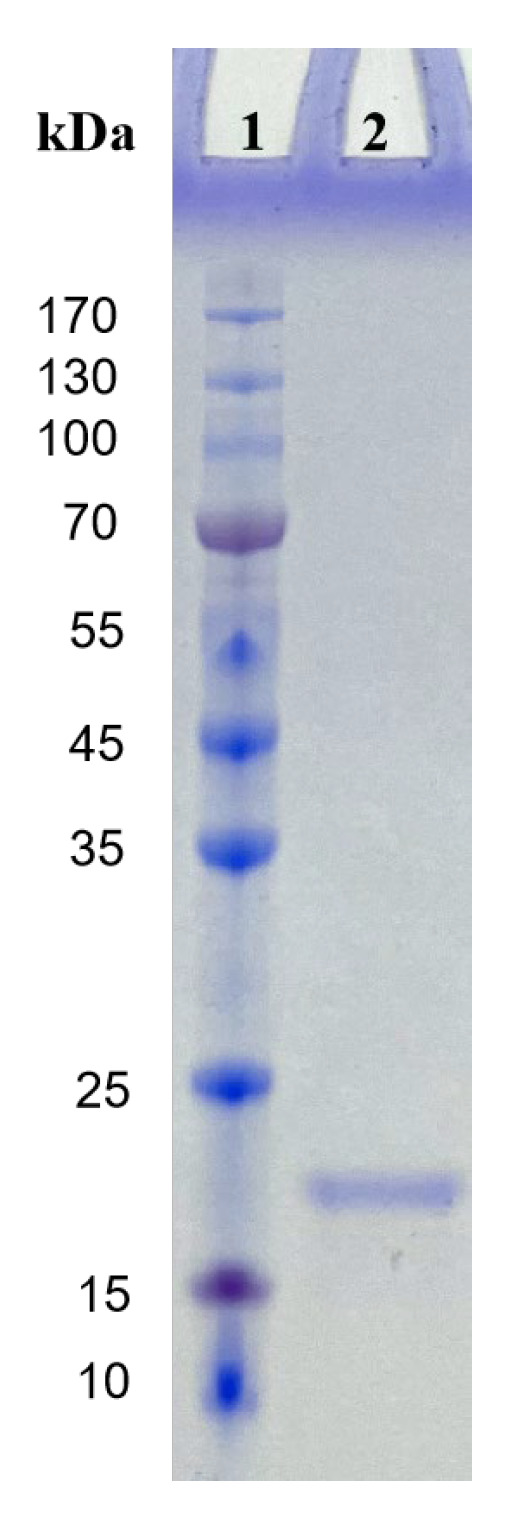
Molecular weight determination by SDS-PAGE under denaturing conditions. Lane 1: molecular weight standards; Lane 2: the purified enzyme.

**Figure 3:**
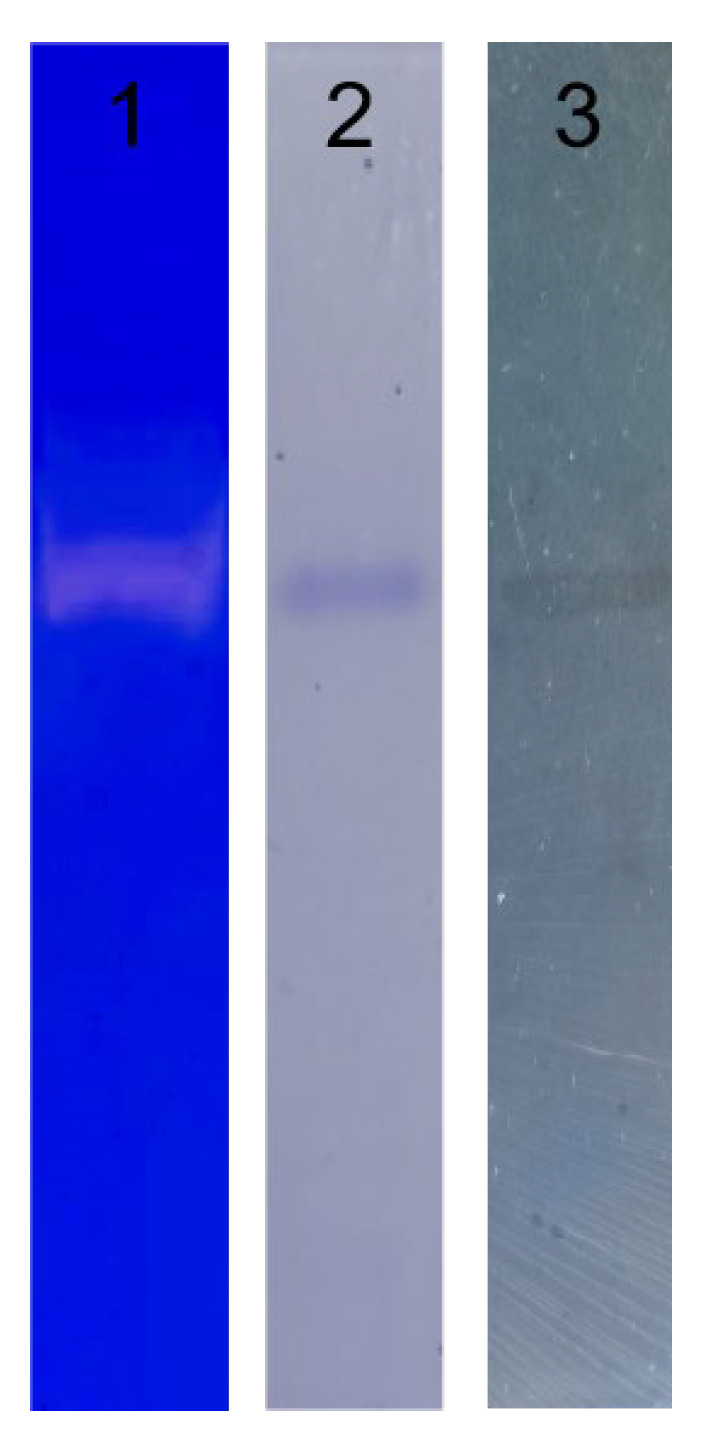
Analysis of the purity of CFE. Lane 1: fibrin zymography; Lane 2: Native-PAGE; Lane 3: the imprint of CFE on a fibrin plate.

The purity of CFE was verified by SDS-PAGE, Native-PAGE, and fibrin zymography electrophoresis. The purified fibrinolytic enzyme from *Coprinus comatus* appeared as a single protein band on SDS-PAGE (Figure 2). It indicated that the fibrinolytic enzyme was a single-subunit protein with a molecular weight of 19.5 kDa. CFE also showed a single band in both Native-PAGE electrophoresis and fibrin zymography electrophoresis (Figure 3), indicating that the sample had reached electrophoretic purity. The band was placed on the fibrin plate and incubated at 37 °C; it was found that transparent areas appeared at the corresponding gel band in the fibrin plate, indicating that the band had fibrinolytic activity and was the target protein. It was worth noting that the molecular weight of CFE was smaller than that of fibrinolytic enzymes reported from mushrooms such as *Boletus pseudocalopus* (63.5 kDa) [26], *Hericium erinaceum* (51 kDa) [27], and *Tremella fuciformis* (38 kDa) [28]. It was similar to fibrinolytic enzymes from *Pleurotus ferulae* (20 kDa) [15], *Tricholoma saponaceum* (18.1 kDa) [29], *Armillaria mellea* (21 kDa) [30], and *Schizophyllum commune* (17 kDa) [31]. Enzymes with smaller molecular weights confer the advantage of lower immunogenicity, making them more suitable for drug or health food development.

### 3.3. Determination of the N-Terminal Amino Acid Sequence

The N-terminal sequences of CFE were determined by Edman degradation. The first nine amino acids of the purified enzyme were A-T-Y-T-G-G-S-Q-T. By analyzing this sequence with the UniProt-BLAST database, we found that a portion of the N-terminal sequence of the purified enzyme shares homology with those of previously reported enzymes (Table 5). It was similar to *Prevotella* sp. (88%, MBP1540255.1), *Mytilus galloprovincialis* (88%, VDI71986.1), *Clostridium saccharoperbutylacetonicum* (89%, WP_015393369.1), and *Prolixibacteraceae bacterium* (89%, HBL77885.1). It is worth noting that the sequence of CFE does not exactly match the N-terminal sequence of any known protease, indicating that it is a novel enzyme.

### 3.4. Effects of Temperature and pH on the Activity of CFE

Temperature exerts a significant impact on enzymes, primarily influencing their stability and susceptibility to thermal denaturation. Additionally, it can affect both the rate of enzymatic reaction and lead to enzyme inactivation. The activity of enzymes is also influenced by pH. Changes in pH not only impact the spatial conformation of enzyme molecules but also affect the ionization and affinity between enzyme and substrate molecules [31,32,33].

**Figure 4:**
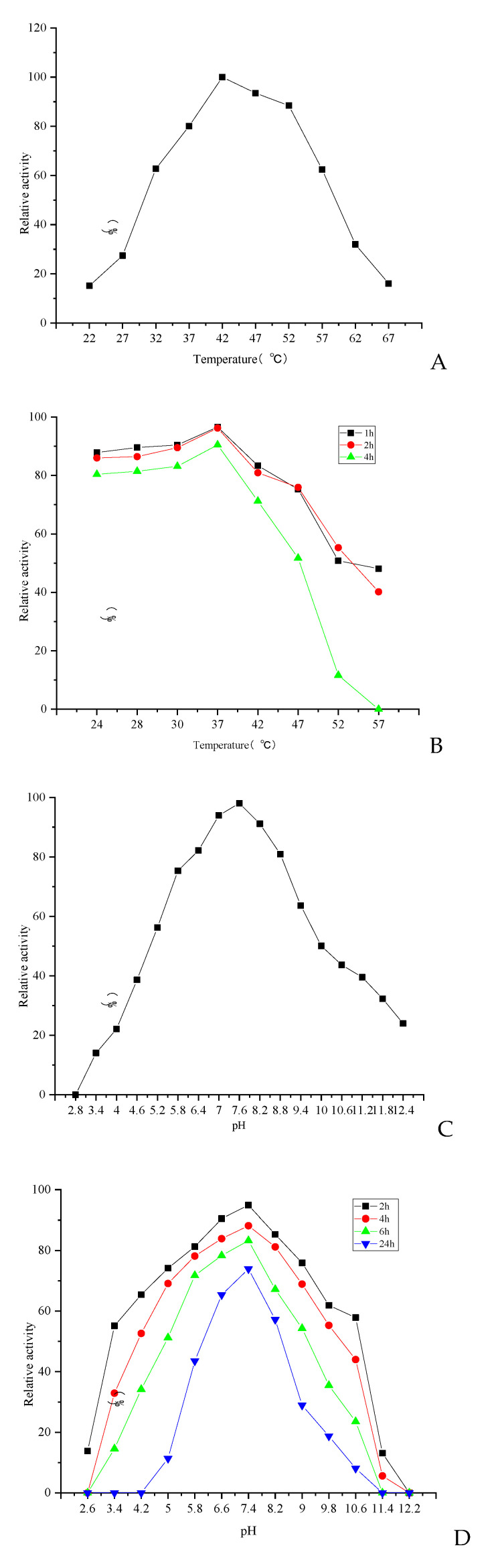
Effects of temperature and pH on fibrinolytic activity of CFE. (**A**) Optimal temperature. (**B**) Temperature stability. (**C**) Optimal pH. (**D**) pH stability.

The optimum temperature, pH, thermal stability, and pH stability of CFE were studied by measuring the residual enzyme activity with the fibrin plate method. The effect of temperature on the fibrinolytic activity of the enzyme was examined within a temperature range of 22 °C to 67 °C, revealing an optimal temperature of 42 °C (Figure 4A). The thermal stability of CFE was measured in the range of 24 °C to 57 °C. The results showed that the enzyme exhibited good thermal stability within the temperature range of 24 °C to 42 °C, with the fibrinolytic activity remaining above 80% after incubation for 1–2 h at these temperatures. Even after a prolonged incubation period of 4 h at these temperatures, the enzyme maintained an activity level exceeding 71.32% (Figure 4B). However, the relative enzyme activity and thermal stability of the enzyme decreased significantly when the temperature exceeded 47 °C. Optimal temperatures for the majority of fibrinolytic enzymes derived from mushrooms typically fall within the temperature range of 20 °C to 60 °C [29,30,31,32,33,34,35]. The optimal temperature of CFE was similar to that of the fibrinolytic enzymes from *Bacillus amyloliquefaciens* GUTU06 [32] and *Coprinopsis atramentaria* [33], which exhibited optimal temperatures of 45 °C and 50 °C, respectively. However, it was higher than that of the fibrinolytic enzymes from *Hericium erinaceum* [27] and *Cordyceps militaris* [13], which exhibited optimal temperatures of 30 °C and 37 °C, respectively.

The effect of pH on fibrinolytic activity was examined at different pH levels. Optimal fibrinolytic activity was observed at pH 7.6 (Figure 4C), which corresponds to the physiological pH of humans. The enzyme exhibited favorable stability within the pH range of 6.6 to 8.2 for 6 h. In this stable pH range, the enzyme activity remained above 80% after 4 h of incubation and still exceeded 71.2% after 6 h. However, following a 24 h incubation period, the residual enzyme activity was observed to be greater than 60.2% (Figure 4D). As already reported, the optimal pH for mushroom fibrinolytic enzymes was within a pH range of 4 to 9, although most of them had an optimal pH between 7 and 7.8 [14,16,29,30,31,32,33,34,35]. Fibrinolytic enzymes, such as those from *Neurospora sitophila* [36] and *Pleurotus ostreatus* [14], showed similar results with an optimal pH value of 7.4. However, it differs from the fibrinolytic enzymes extracted from *Flammulina velutipes* [10] and *Pleurotus eryngii* [16], as they had optimal pH values of 5.0 and 6.0.

### 3.5. Effects of Metal Ions, Protease Inhibitors, and Some Reagents on Fibrinolytic Activity of CFE

Metal ions as components of enzymes or activators affect enzymatic reactions. The fibrinolytic activity of CFE was influenced by the presence of metal ions. It was found that Zn^2+^, K^+^, Ca^2+^, Mn^2+^, and Mg^2+^ stimulated fibrinolytic activity, whereas Fe^2+^, Fe^3+^, Al^2+^, and Ba^2+^ inhibited fibrinolytic activity as shown by the residual enzyme activity (Table 6). The results indicated that Zn^2+^, K^+^, Ca^2+^, Mn^2+^, and Mg^2+^ might serve as the active central components or activators of CFE. The results were similar to those previously reported fibrinolytic enzymes, such as the enzyme from *Pleurotus ostreatus* [14], which exhibited activation in the presence of Ca^2+^ and K^+^ while being inhibited by Fe^2+^ and Fe^3+^. The protease with fibrinolytic activity from *Coprinopsis atramentaria* [33] was activated by Mg^2+^ and inhibited by Al^2+^, whereas the fibrinolytic enzyme from *Hericium erinaceum* [27] showed activation in the presence of Ca^2+^, Mg^2+^, and Mn^2+^ but inhibition in the presence of Cu^2+^ and Fe^2+^.

Protease inhibitors can interact with specific groups on the active site of the protease, leading to a reduction in protease activity without inducing denaturation of the enzyme proteins. According to the inhibitor specificity of the protein, PMSF is a typical inhibitor of both serine proteases and cysteine proteases, effectively inhibiting the activity of serine proteases as well as sulfhydryl protease. EDTA inhibits metalloproteinases, aprotinine inhibits serine proteases, TPCK inhibits chymotrypsin, SBTI is a soybean trypsin inhibitor, and pepstatin is a pepsin inhibitor [28,31]. The activity of fibrinolytic enzymes was examined in the presence of some protease inhibitors (Table 7). The enzyme was completely inhibited by 1–10 mM EDTA, and it was also partially inhibited by PMSF and SBTI, resulting in inhibitions of 12.98% and 47.19%, respectively, at a concentration of 10 mmol/L. The enzyme activity was mildly inhibited by a high concentration of aprotinin. It was implied that CFE might be a serine-like metalloproteinase, potentially containing active centers similar to those found in soybean trypsin. The effects of TPCK and pepstatin on the enzyme activity were found to be insignificant. The observation suggested that the enzyme did not have a similar active center to chymotrypsin and pepsin. Fibrinolytic enzymes from *Tremella fuciformis* [28] and *Schizophyllum commune* [31] were also found to be inhibited by EDTA. The fibrinolytic enzyme produced by *Bacillus licheniformis* KJ-31 [37] was completely inhibited by a low concentration of PMSF, while EDTA did not affect its activity.

The impact of some protective agents, protein denaturants, and organic solvents on the enzyme activity was investigated (Table 8). Based on the relative enzyme activity, it was observed that SDS and urea exhibited inhibitory effects on fibrinolytic activity, suggesting that these reagents could potentially cause denaturation of the enzyme. The fibrinolytic activity was not significantly affected by reduced glutathione, oxidized glutathione, cysteine, or β-mercaptoethanol. These findings indicated that the presence of a sulfhydryl group did not have a significant impact on the activity of CFE. The activity of the enzyme was slightly inhibited by BSA, and acetone also had an inhibitory effect on enzyme activity. Gelatin, peptone, and glycerin were found to enhance the activity of the enzyme, suggesting their protective effect on CFE.

### 3.6. Plasminogen Activator Activity of CFE

**Figure 5:**
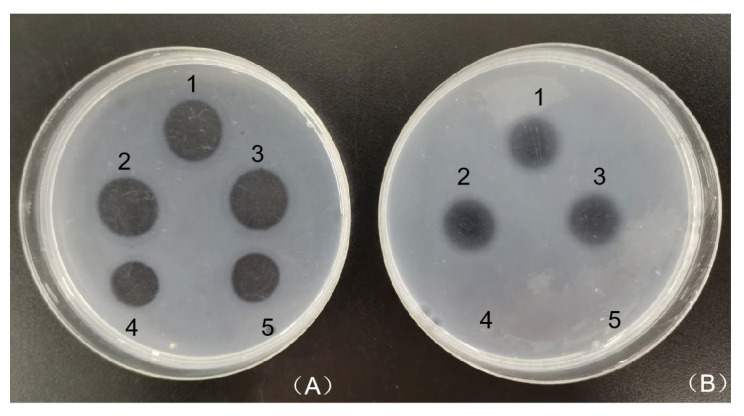
Analysis of plasminogen activation by CFE on (**A**) plasminogen-positive fibrin plate and (**B**) plasminogen-negative fibrin plate. Circles 1–3 represent CFE; circles 4–5 represent urokinase.

The degradation of thrombus by fibrinolytic enzymes involves two pathways: one directly degrades coagulation, and the other one indirectly degrades fibrin by activating plasminogen into plasmin. The plasminogen activator activity of CFE was examined on plasminogen-negative and plasminogen-positive fibrin plates. The clarity and size of the lysis zones in plasminogen-negative and plasminogen-positive fibrin plates were significantly different (Figure 5A,B). The hydrolytic circles on the plasminogen-positive fibrin plate were larger and clearer than those on the plasminogen-negative fibrin plate, while urokinase only showed lysis zones on the plasminogen-positive fibrin plate, indicating that CFE could not only directly degrade fibrin, but also activated the conversion of plasminogen into plasmin. These results suggested that CFE could act as a plasminogen activator. The enzyme was similar to fibrinolytic enzymes from *Cordyceps militaris*, *Agrocybe aegerita*, and *Sipunculus nudus* [13,23,38], which exhibited dual fibrinolytic effects. In contrast, the fibrinolytic enzyme from *Lyophyllum shimeji* [39] could only indirectly contribute to the dissolution of fibrin.

### 3.7. Effects of Simulated Gastric and Blood Environment on the Fibrinolytic Activity of CFE

**Figure 6:**
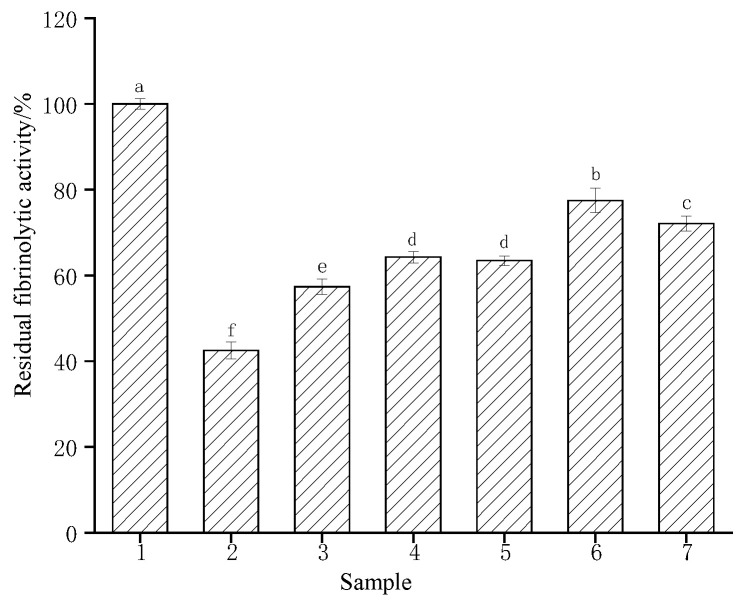
Effect of simulated gastric and blood environments on the CFE. Sample 1, CFE in water (control); sample 2, CFE in artificial gastric juice; sample 3, CFE in gastric juices with a pH of 7.4; sample 4, CFE in artificial gastric juice and broth; sample 5, CFE in artificial gastric juice and saccharose; sample 6, CFE in artificial gastric juice, saccharose, and protein broth; sample 7, CFE in Locke solution. Different letters indicate significant differences (*p* < 0.05).

Research the tolerance of CFE to some human environments, the residual enzyme activity was measured in simulated human gastric juices and blood (Table 1 and Figure 6). The fibrinolytic activity of CFE was inhibited by gastric juice and showed 42.5% residual activity in artificial gastric juice after a 4 h incubation period (gastric emptying time). The enzyme activity remained at 57.36% after a 4 h incubation when the pH of simulated gastric juice was adjusted to 7.4, indicating that the fibrinolytic activity of CFE might be inhibited in a short time but not deactivated. When broth or sucrose was added to gastric juice as a protective agent, the residual enzyme activities were 64.27% and 63.47%, respectively, after a 4 h incubation period. However, when both carbohydrates and broth were present simultaneously, the residual enzyme activity was 77.49%, indicating that the presence of both carbohydrates and proteins exerted a synergistic protective effect on the activity of CFE. Therefore, taking the enzyme after meals could help maintain some of its activity. However, the absorption process in the small intestine further reduced the activity. The enzyme also exhibited tolerance to the simulated blood environment and maintained a relative activity of 72.11% after being incubated in Locke solution for 4 h. Based on the above results, the enzyme has demonstrated its ability to withstand both the physiological conditions of the digestive system and the human blood environment. Therefore, it is recommended to be taken after a meal or administered intravenously. Similarly, fibrinolytic enzymes purified from *Agrocybe aegerita* [23] and *Cordyceps militaris* [40] exhibited inhibitory effects on enzyme activity in gastric juices, while protein solution and saccharose exerted a protective effect on enzyme activity, resulting in the maintenance of approximately 60% residual enzyme activity through intravenous administration.

### 3.8. Analysis of Thrombin-like Activity of CFE

**Figure 7:**
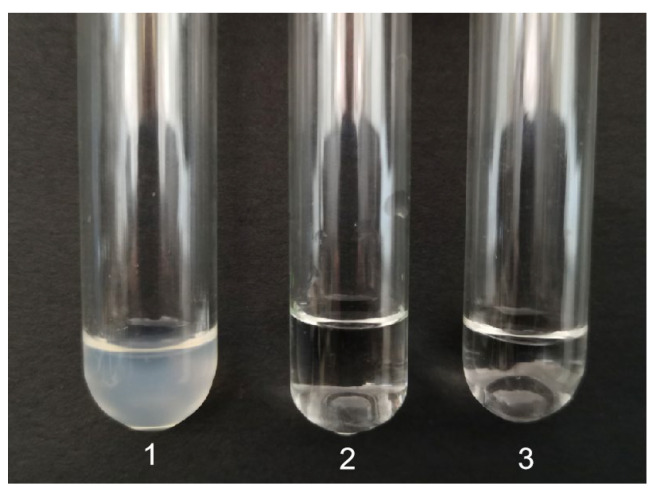
Analysis of thrombin-like activity of CFE. 1, control fibrin clot (human blood fibrinogen and human thrombin); 2, CFE, human blood fibrinogen, and human thrombin; 3, human blood fibrinogen and CFE.

The thrombin-like activity of CFE was analyzed by comparing clot formation in different mixtures (Table 2). The results are shown in Figure 7. The formation of a blood clot was induced in test tube 1 by mixing human thrombin and human blood fibrinogen, serving as a positive control. The combination of CFE and human blood fibrinogen in test tube 2 displayed a distinct absence of fibrin clots, even after the addition of human thrombin to the mixture. The findings revealed that CFE disrupted the structure of fibrinogen, thereby impeding the conversion into fibrin by thrombin. The absence of fibrin clot formation in the mixture of human blood fibrinogen and CFE, as observed in test tube 3, indicates that CFE did not have thrombin-like activity. Li et al. [23] also observed a similar result for a fibrinolytic enzyme from *Agrocybe aegerita*. The findings suggest that CFE has the potential to serve as an effective anticoagulant for the prevention of fibrin clot formation.

### 3.9. In Vitro Fibrinolytic Effect of CFE on Blood Clots

The fibrinolytic effect of CFE in vitro was evaluated with whole blood clots of healthy volunteers. The dissolution rate of CFE in blood clots was determined at different time intervals. The results, presented in Table 9, demonstrated the ability of CFE to dissolve blood clots with a significant increase in dissolution rate over time. The rate of blood clot dissolution exceeded 50% within 150 min and surpassed 80% within a period of 24 h. The results of in vitro clot lysis indicated that CFE exhibits significant thrombolytic activity. CFE exhibited similar activity to fibrinolytic enzymes from *Cochliobolus hawaiiensis* [41] and *Bacillus subtilis* DC33 [42], which have previously been reported to possess the ability to dissolve blood clots.

### 3.10. Fibrin(ogen)olytic Activity of CFE

**Figure 8:**
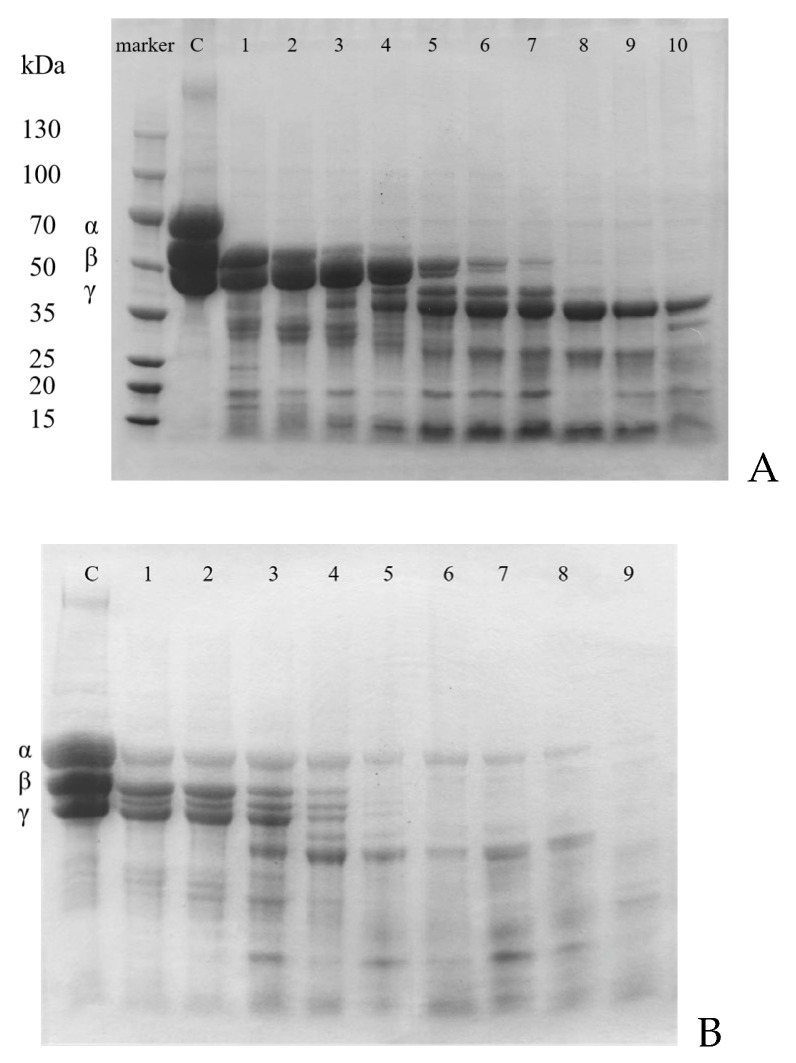
Cleavage pattern of fibrin(ogen) by CFE; (**A**) SDS-PAGE analysis of human blood fibrinogen hydrolyzed by CFE. Lane C, control; Lanes 1–10, degradation pattern of fibrinogen at different time intervals of 1 min, 5 min, 15 min, 30 min, 1 h, 1.5 h, 2 h, 3 h, 4 h, and 5 h, respectively. (**B**) SDS-PAGE analysis of human fibrin hydrolyzed by CFE; Lane C, control; Lanes 1–9, degradation pattern of fibrin at different time intervals of 1 min, 5 min, 15 min, 30 min, 1 h, 2 h, 3 h, 4 h, and 5 h, respectively.

The fibrin(ogen)olytic activity of CFE and the cleavage pattern of fibrin(ogen) chains were analyzed (Figure 8A,B). Fibrinogen and fibrin both consist of Aα, Bβ, and γ chains. The degradation mode of fibrinogen and fibrin was confirmed by analyzing the disappearance order of those bands. The fibrinogen degradation activity of CFE was verified by SDS-PAGE (Figure 8A). Under the action of CFE, the Aα chain of fibrinogen was degraded first, followed by the Bβ chain and γ chain. It was noteworthy that the Aα chain was degraded by CFE within 1 min, whereas the Bβ chain was degraded within 1 h, and the γ chain underwent slow degradation for 3 h. According to previous reports, the fibrinolytic enzyme from *Hericium erinaceum* [27] caused rapid degradation of the Aα chain of fibrinogen and slower degradation of the γ chain, but it did not hydrolyze the Bβ chain of fibrinogen. According to the study conducted by Kim et al. [43], it was observed that the fibrinolytic enzyme derived from *Cordyceps militaris* initially targeted the Aα chains of fibrinogen for hydrolysis, followed by subsequent hydrolysis of the γ and Bβ chains. A fibrinolytic enzyme, MA-1, purified from *Mycoleptodonoides aitchisonii* [44] first hydrolyzed the Aα chain of fibrinogen, then the γ and Bβ chains.

The cleavage effects of CFE on fibrin (Figure 8B) were also investigated. The degradation patterns of fibrin were different from those of fibrinogen. The β and γ chains were simultaneously hydrolyzed, resulting in the complete degradation of both chains within 2 h of incubation. The Aα chain of fibrin was hydrolyzed quite slowly and was completely degraded in 5 h. Its degradation pattern differed from that of fibrinolytic enzymes found in *Flammulina velutipes* [10] and *Pleurotus ostreatus* [14], which could sequentially degrade the Aα, Bβ, and γ chains of fibrin. The Aα chain was rapidly hydrolyzed by the fibrinolytic enzyme derived from *Cordyceps militaris* [13] fruiting bodies, while the hydrolysis of the γ chain and Bβ chain was slow.

### 3.11. Effects of CFE on Some Blood Proteins

**Figure 9:**
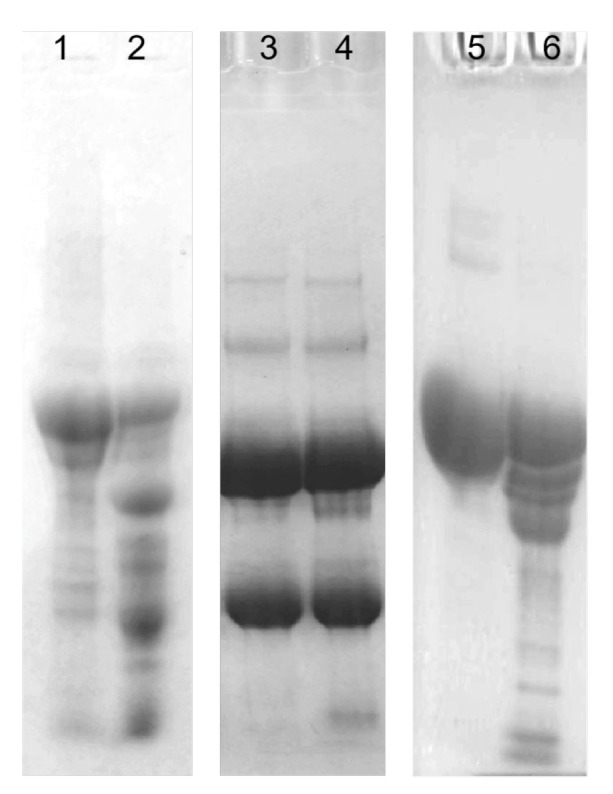
Effects of CFE on some blood proteins. Lanes 1, 3, and 5 represent human thrombin, IgG, and human serum albumin (HSA), respectively. Lanes 2, 4, and 6 represent human thrombin, IgG, and HSA incubated with CFE, respectively.

The effect of CFE on some typical human blood proteins such as human serum albumin (has), immunoglobulin G (IgG), and human thrombin was studied by SDS-PAGhasHSA plays a crucial physiological role in the body, serving a pivotal function in enlarging blood volume and maintaining plasma osmotic pressure. IgG accounts for about 70% of human plasma globulin and has the dual role of immune regulation and immune enhancement. As a coagulation factor, thrombin plays a key role in the process of thrombosis. The blood proteins were incubated with CFE for 4 h, and the degradation patterns are shown in Figure 9. The enzyme exhibited slight hydrolysis of Ighasnd HSA, indicating that it has some adverse effects on the immune system. CFE also exhibited partial degradation of human thrombin, thereby demonstrating its potential to inhibit fibrin clot formation and consequently prevent thrombosis. Liu et al. [13] found that the enzyme from *Cordyceps militaris* could partially hydrolyze human thrombin and serum albumin but had no significant effect on IgG.

### 3.12. Analysis of Anticoagulation Activity of CFE In Vitro

The assessment of anticoagulant activity involves evaluating the coagulation screen and the inhibitory effect of CFE on blood clot formation in vitro. The coagulation screen, which includes activated partial thromboplastin time (APTT), prothrombin time (PT), and prothrombin activity (PA), thrombin time (TT), and fibrinogen (FIB) assays, is a crucial clinical diagnostic index commonly employed for identifying fibrinolysis or coagulation disorders in vitro [45,46,47]. APTT and PT are used to measure the activity of the extrinsic and intrinsic pathways of the clotting cascade, respectively. PA can more precisely reflect the activity of coagulation factors. TT represents the time required for the conversion of fibrinogen into fibrin, and FIB indicates the concentration of fibrinogen in a sample.

**Figure 10:**
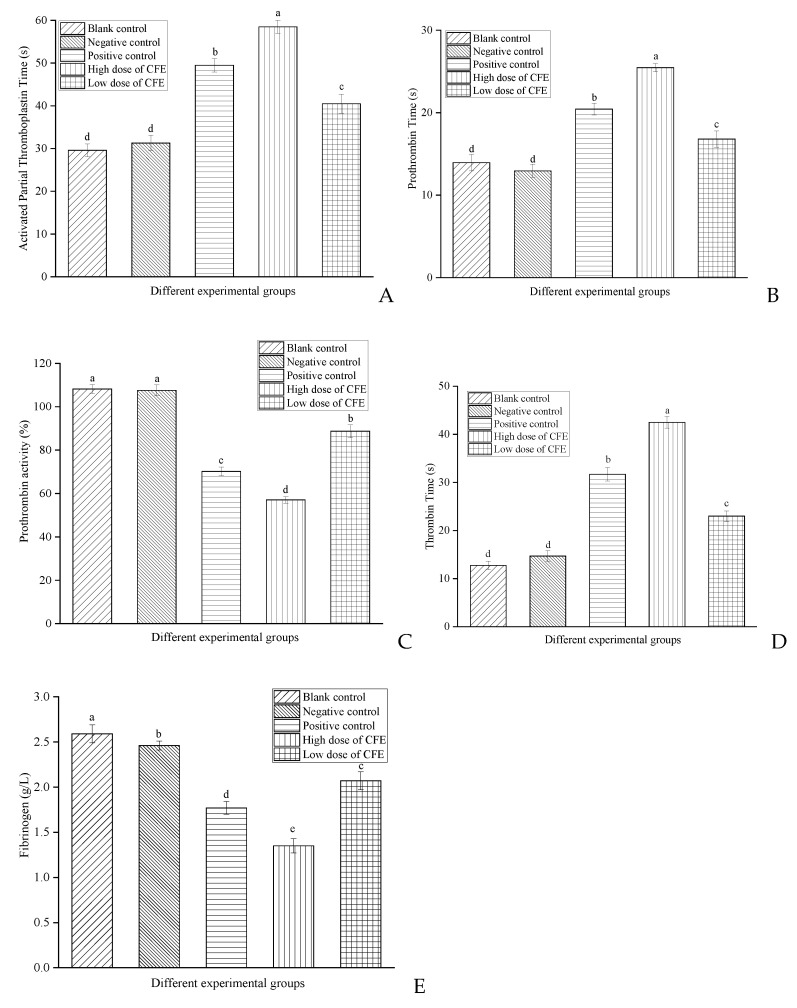
Effects of CFE on coagulation index in vitro. The levels of (**A**) APTT, (**B**) PT, (**C**) PA, (**D**) TT, and (**E**) FIB in different experimental groups. Different letters indicate significant differences (*p* < 0.05).

CFE was incubated with the plasma of healthy volunteers at 37 °C for 3 min, followed by the detection of its impact on coagulation screening indexes using an automatic coagulation analyzer. The results are shown in Figure 10. The results demonstrated that, in comparison to the blank group and negative control group, both the high- and low-dose groups of CFE exhibited significantly increased levels of APTT, PT, and TT and simultaneously decreased levels of FIB and PA. Moreover, the high-dose CFE group exhibited a more pronounced effect compared to that of the positive control group, while the effect observed in the positive control group surpassed that of the low-dose CFE group. On the one hand, the increase in the APTT and PT levels (Figure 10A,B) indicated that CFE inhibited the coagulation cascade in both the intrinsic and extrinsic pathways of coagulation. The speculation was that CFE might impede coagulation factors XII, XI, Ⅴ, and Ⅶ in both the endogenous and exogenous coagulation pathways, hinder the activation of coagulation factor X to Xa, inhibit the conversion of prothrombin to thrombin, and result in a reduction in PA levels (Figure 10C). Meanwhile, Section 3.11 demonstrated that CFE could hydrolyze thrombin. As a result, a reduction in thrombin leads to an extended conversion time for fibrinogen into fibrin, which was reflected in the improvement of TT levels (Figure 10D). On the other hand, as depicted in Figure 10E, CFE could reduce the content of FIB. This result also confirmed the experimental results of Section 3.10, which demonstrated that CFE possesses the capacity to hydrolyze and disrupt the structural integrity of fibrinogen, thereby impeding its conversion into fibrin and resulting in a reduction in FIB content. The experimental results of the coagulation index demonstrate that CFE could serve as a potent plasma anticoagulant or fibrinolytic agent to prevent thrombosis effectively. However, administering anticoagulants requires a delicate equilibrium between under- and over-treatment. Choi et al. [15] found that the fibrinolytic enzyme from *Pleurotus ferulae* significantly prolonged the levels of APTT and PT in vitro. Yang et al. [45] discovered that the protease with fibrinolytic activity could increase the levels of APTT and PT in plasma. The fibrinolytic enzyme from *Petasites japonicus* [46] significantly increased the APTT level but had no notable impact on PT.

**Figure 11:**
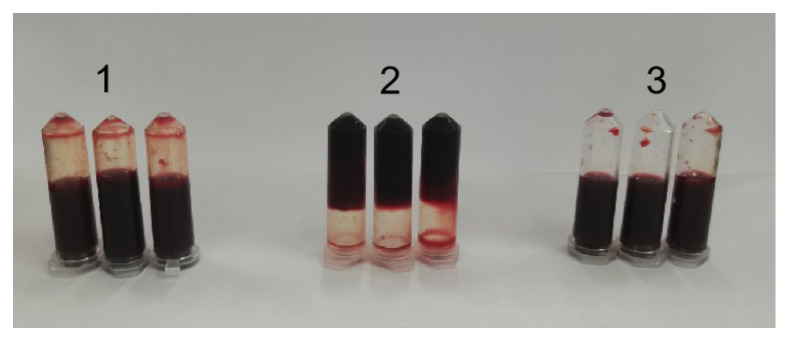
Anticoagulant activity of CFE. (1) Plasma and heparin; (2) plasma and saline; (3) plasma and CFE.

The anticoagulant property of CFE in vitro was also researched. With normal saline as a negative control and heparin sodium as a positive control, the coagulation of blood in different groups of centrifuge tubes was observed. The coagulation time of fresh blood was 292 s, and the coagulation time of blood in normal saline was 768 s. As shown in Figure 11, samples with heparin and CFE did not coagulate even after 4 h. After incubation for 24 h, the blood in the heparin group became viscous and its flow state was blocked, whereas the blood in the CFE group remained non-coagulated and retained its free-flowing state. The results showed that CFE could be used as an anticoagulant, and its anticoagulant activity was slightly better than heparin.

## 4. Conclusions

CFE, a novel fibrinolytic enzyme, was found in *Coprinus comatus*. The purified enzyme was composed of a single subunit with a specific activity of 3619 U/g, and its molecular weight was 19.5 kDa. The optimum temperature and pH of the enzyme were 42 °C and 7.6, respectively. The fibrinolytic activity was activated by the metal ions Zn^2+^, K^+^, Ca^2+^, Mn^2+^, and Mg^2+^ but inhibited by Fe^2+^, Fe^3+^, Al^2+^, and Ba^2+^. CFE could hydrolyze human fibrinogen and fibrin. It was both a direct hydrolyzer of fibrin and a plasminogen activator. CFE exhibited a slight hydrolytic effect on thrombin and demonstrated anticoagulant activity in vitro. Furthermore, it could increase APTT, PT, and TT levels while reducing FIB and PA levels, thereby inhibiting blood clotting. Based on the above findings, CFE has great potential in the prevention and treatment of thrombosis.

## Figures and Tables

**Table 1:** The experimental method of simulated blood and gastric environment.

Group	Composition
1	100 μL H_2_O, 50 μL CFE
2	100 μL gastric juice, 50 μL CFE
3	100 μL gastric juice with a pH of 7.4, 50 μL CFE
4	50 μL gastric juice, 50 μL of broth, 50 μL CFE
5	50 μL gastric juice, 50 μL 10% saccharose, 50 μL CFE
6	50 μL gastric juice, 25 μL broth, 25 μL 10% saccharose, 50 μL CFE
7	100 μL Locke solution, 50 μL CFE

**Table 2:** The experimental method for the analysis of thrombin-like activity.

Group	Composition
1	Human blood fibrinogen (10 mg/mL), human thrombin (200 U/mL)
2	Human blood fibrinogen (10 mg/mL), human thrombin (200 U/mL), CFE (72 U/mL)
3	Human blood fibrinogen (10 mg/mL), CFE (72 U/mL)

**Table 3:** The experimental method for the analysis of anticoagulant activity.

Group	Composition
Blank control group	500 μL PPP
Negative control group	500 μL PPP, 500 μL normal saline
Positive control group	500 μL PPP, 500 μL heparin sodium (30 U/mL)
CFE low-dose group	500 μL PPP, 500 μL CFE (10 U/mL)
CFE high-dose group	500 μL PPP, 500 μL CFE (30 U/mL)

**Table 4:** Purification steps of the fibrinolytic enzyme from *Coprinus comatus*.

Purification Steps	Volume	Protein	Activity	Recovery	Specific Activity	Purification Fold
	mL	mg	U	%	U·mg^−1^	
Crude enzyme	200.00	1684.00	25,286.00	100.00	15.02	1.00
60% (NH_4_)_2_SO_4_	50.00	293.00	21,847.00	86.40	74.56	4.97
Octyl-FF	69.00	40.02	18,415.41	72.83	460.16	30.65
G-25	120.00	27.60	16,098.00	63.66	583.26	38.84
SP-HP	28.00	4.48	6943.44	27.46	1907.54	127.04
Source 15PHE	7.00	0.69	2533.30	10.02	3619.00	241.02

**Table 5:** Comparison of N-terminal sequence of CFE.

Source	Position of First Amino Acid	Sequence	Identity (%)	Accession Number
*Coprinus comatus*		ATYTGGSQT		This study
*Prevotella* sp.	90	TYTGGSQT	88%	MBP1540255.1
*Mytilus galloprovincialis*	536	TYTGGSQT	89%	VDI71986.1
*Clostridium saccharoperbutylacetonicum*	205	ATYTGGAQT	89%	WP_015393369.1
*Prolixibacteraceae bacterium*	321	ATYTGGTQT	88%	HBL77885.1

**Table 6:** Effects of some metal ions on the fibrinolytic activity of CFE.

Metal Ions	Residual Fibrinolytic Activity (%)
Control	100
Zn^2+^	154.51 ± 2.21
Fe^2+^	11.75 ± 0.53
Cu^2+^	95.74 ± 0.93
Fe^3+^	40.45 ± 0.64
K^+^	110.89 ± 3.63
Ca^2+^	173.20 ± 1.17
Na^+^	97.85 ± 2.93
Mn^2+^	146.33 ± 3.01
Al^2+^	30.28 ± 0.87
Mg^2+^	108.50 ± 3.16
Ba^2+^	76.18 ± 3.27

**Table 7:** Effects of protease inhibitors on the fibrinolytic activity of CFE.

Inhibitor	Concentration (mmol/L)	Residual Fibrinolytic Activity (%)
Control		100
Aprotinine	10	85.53 ± 1.27
	5	97.89 ± 2.26
	2.5	104.88 ± 1.63
	1	107.03 ± 1.81
PMSF	10	12.98 ± 2.11
	5	24.26 ± 1.82
	2.5	30.46 ± 3.21
	1	39.12 ± 2.93
TPCK	10	87.21 ± 1.39
	5	90.03 ± 2.22
	2.5	105.76 ± 2.31
	1	98.11 ± 1.97
Pepstatin	10	92.37 ± 1.34
	5	94.82 ± 1.41
	2.5	97.20 ± 1.68
	1	98.32 ± 2.08
EDTA	10	0.00
	5	0.00
	2.5	0.00
	1	0.00
SBTI	10	47.19 ± 2.36
	5	58.42 ± 1.83
	2.5	79.46 ± 1.96
	1	91.67 ± 2.21

**Table 8:** Effects of some reagents on the fibrinolytic activity of CFE.

Reagent	Concentration	Residual Fibrinolytic Activity (%)
Control		100
Cysteine	20 mmol/L	97.85 ± 3.27
Reduced glutathione	5 mmol/L	92.62 ± 2.83
Oxidized glutathione	5 mmol/L	96.34 ± 2.75
β-mercaptoethanol	0.50%	97.02 ± 2.48
Peptone	1%	103.08 ± 2.74
Gelatin	1%	149.91 ± 1.29
Bovine serum albumin	1%	80.61 ± 2.39
Acetone	10%	89.95 ± 1.86
Glycerol	10%	168.78 ± 1.95
SDS	0.30%	4.64 ± 0.22
Urea	8 mol/L	45.33 ± 3.08

**Table 9:** Analysis of dissolution of blood clots by CFE at different time intervals.

Time	Weight (g)	Dissolution Rate (%)
0	0.5058 ± 0.0062	0.00
10 min	0.4713 ± 0.0058	6.82
20 min	0.4082 ± 0.0047	19.30
40 min	0.3647 ± 0.0039	27.90
60 min	0.3329 ± 0.0042	34.18
90 min	0.3183 ± 0.0028	37.07
120 min	0.2803 ± 0.0036	44.58
150 min	0.2436 ± 0.0048	51.84
180 min	0.2229 ± 0.0052	55.93
12 h	0.1728 ± 0.0041	65.84
24 h	0.0882 ± 0.0032	82.56

## Data Availability

The original contributions presented in the study are included in the article, further inquiries can be directed to the corresponding author.
